# A fast and effective determination of the biodistribution and subcellular localization of fluorescent immunoliposomes in freshly excised animal organs

**DOI:** 10.1186/s12896-017-0327-8

**Published:** 2017-01-18

**Authors:** Felista L. Tansi, Ronny Rüger, Ansgar M. Kollmeier, Claudia Böhm, Roland E. Kontermann, Ulf K. Teichgraeber, Alfred Fahr, Ingrid Hilger

**Affiliations:** 1 Institute of Diagnostic and Interventional Radiology, Experimental Radiology, Jena University Hospital, Am Klinikum 1, 07747 Jena, Germany; 2 Department of Pharmaceutical Technology, Friedrich-Schiller-University Jena, Lessingstrasse 8, 07743 Jena, Germany; 3 Institute of Cell Biology and Immunology, University Stuttgart, Allmandring 31, 70569 Stuttgart, Germany

**Keywords:** Biodistribution studies, Fluorescence and autofluorescence imaging, Molecular targeting, Liposomes

## Abstract

**Background:**

Preclinical research implementing fluorescence-based approaches is inevitable for drug discovery and technology. For example, a variety of contrast agents developed for biomedical imaging are usually evaluated in cell systems and animal models based on their conjugation to fluorescent dyes. Biodistribution studies of excised organs are often performed by macroscopic imaging, whereas the subcellular localization though vital, is often neglected or further validated by histological procedures. Available systems used to define the subcellular biodistribution of contrast agents such as intravital microscopes or ex vivo histological analysis are expensive and not affordable by the majority of researchers, or encompass tedious and time consuming steps that may modify the contrast agents and falsify the results. Thus, affordable and more reliable approaches to study the biodistribution of contrast agents are required. We developed fluorescent immunoliposomes specific for human fibroblast activation protein and murine endoglin, and used macroscopic fluorescence imaging and confocal microscopy to determine their biodistribution and subcellular localization in freshly excised mice organs at different time points post intravenous injection.

**Results:**

Near infrared fluorescence macroscopic imaging revealed key differences in the biodistribution of the respective immunoliposomes at different time points post injection, which correlated to the first-pass effect as well as the binding of the probes to molecular targets within the mice organs. Thus, a higher accumulation and longer retention of the murine endoglin immunoliposomes was seen in the lungs, liver and kidneys than the FAP specific immunoliposomes. Confocal microscopy showed that tissue autofluorescence enables detection of organ morphology and cellular components within freshly excised, non-processed organs, and that fluorescent probes with absorption and emission maxima beyond the tissue autofluorescence range can be easily distinguished. Hence, the endoglin targeting immunoliposomes retained in some organs could be detected in the vascular endothelia cells of the organs.

**Conclusions:**

The underlying work represents a quick, effective and more reliable setup to validate the macroscopic and subcellular biodistribution of contrast agents in freshly excised animal organs. The approach will be highly beneficial to many researchers involved in nanodrug design or in fluorescence-based studies on disease pathogenesis.

## Background

In the majority of diseases, molecular alterations precede detectable pathological changes by various durations, which can range from weeks to years. Such molecular events and changes help in prediction, diagnosis and therapy of diseases. Therefore, molecular imaging, which is defined as the non-invasive real time visualization of biochemical events at the cellular and molecular level within living cells, tissues and/or whole organisms [1–3] holds an influential position in medicine. Thus, molecular imaging has been implemented in a wide field of biomedical research on drug discovery [4, 5], disease pathogenesis and is of vital importance in nuclear medicine, amongst others [6]. In preclinical research, molecular imaging is applied to study disease pathogenesis, drug efficacy and diagnostic properties of contrast agents and molecular tracers. Such researches often rely on fluorescent agents that can be attached to drugs, tracers and other non-fluorescent contrast agents in order to non-invasively monitor their properties and biodistribution by fluorescence detection [5]. For instance fluorescence imaging is exploited to characterize contrast agents aimed for applications in positron emission tomography (PET) [7] or magnetic resonance imaging (MRI) [8]. Due to the limited penetration depth of light, fluorescence imaging is more feasible in diagnostic imaging of superficial diseases such as rheumatoid arthritis [9], skin, head and neck and breast cancers and for endoscopic imaging of colon cancers as well as intraoperative setups where the surgeon directly visualizes diseased tissues in real time [10]. Hence, fluorescence imaging is more widely applied in drug development and studies on disease pathogenesis, and as well in theranostic approaches, whereby the dyes which serve as therapeutics are encapsulated in the core of lipidic nanoparticles, as was recently demonstrated by Anikeeva et al. [11]. In such preclinical studies, different criteria are applied to evaluate the suitability of molecular contrast agents or targeted therapeutic drugs for future applications in humans. Besides the stability and specificity of the molecular probes, their biocompatibility and suitable clearance is vital. Therefore, many preclinical animal trials include biodistribution experiments, whereby the fluorescence signals of the drugs or contrast agents within excised organs are monitored ex vivo*.* In this respect, several reports demonstrate macroscopic evidence for the biodistribution of fluorescent probes and contrast agents in organs involved in probe degradation and elimination, such as the liver, kidneys and gastro intestinal tract [12]. However, few reports pinpoint the cells and compartments involved in the accumulation of these probes within the organs. Considering that the subcellular localization or longer retention of drugs and contrast agents in some organs may pose adverse side effects [13, 14], it is relevant to include different exposure time points and also to pinpoint the subcells involved in the biodistribution of the said contrast agents or drugs. Model systems for example intravital microscopes permit kinetic studies of probes and enable visualization of their subcellular localization in the organs in real time [15]. However, most instruments which permit these studies are bulky, expensive and demand expertise in handling. Furthermore, the studies concentrate on a single organ or area at a time, whereas a lot of information in the other organs is not addressed. Ex vivo histological analyses can detect the subcellular localization of probes within organs. This however, involves microscopic imaging of tissue sections and demands conservation and processing of the tissues. The steps involved are tedious, time consuming and also relatively expensive. Furthermore, conservation and processing can lead to loss, or modification of the contrast agents being addressed, resulting in unreliable or contradictory results in some cases.

We therefore looked for alternative cost-effective setups to effectively image and correlate the macroscopic distribution and subcellular localization of fluorescent probes in freshly isolated organs. Using a simple confocal microscopy setup to image freshly excised organs, we could demonstrate the feasibility of determining the subcellular localization of contrast agents and correlate this with results acquired by macroscopic imaging. Hereby, tissue autofluorescence which originate from water, hemoglobin, flavins collagens and many other pigments was exploited in defining several organs / tissue structures as demonstrated earlier [16]. The tissue fluorophores absorb and emit light at different wavelengths which lie beyond the near-infrared (NIR) optical window (650 nm - 900 nm) [17]. Hence, fluorescent probes which absorb and emit at these NIR wavelengths can be conveniently detected. A bulk of preclinical and clinical applications therefore utilizes NIR fluorescent (NIRF) dyes with absorption and emission wavelengths between 650 nm and 900 nm as the source of contrast [18–20]. In the underlying research, the biodistribution and subcellular localization of two liposomal formulations were elucidated. The liposomes were encapsulated with a high concentration of the NIRF dye, DY-676-COOH (excitation /emission: 674 nm /699 nm) and conjugated to single chain antibody fragments (scFv) directed to either fibroblast activation protein (FAP) which is overexpressed on tumor associated fibroblasts of 90% of tumors but not healthy tissues [21], or to endoglin, which is overexpressed on some tumor cells and the majority of tumor neovasculature [22]. We demonstrate that confocal microscopic imaging of freshly excised organs can detect the subcellular localization of fluorescent probes, which could be correlated to the observations made by macroscopic imaging. Hence, characteristic differences were detected in the distribution and subcellular localization of the liposome formulations over time post injection. The results expose the relevance of combining microscopic imaging with macroscopic imaging in order to make reliable conclusions about the biodistribution and related clearance of molecular optical imaging agents. Taken together, the approach is fast, easy to accomplish and represents a reliable and cost effective exploitation of the tissue autofluorescence to substantiate the subcellular localization and biodistribution of imaging probes and fluorescent therapeutics.

## Methods

### Preparation and physicochemical characterization of immunoliposomes

A detailed description of the preparation and physical characterization of the ligand targeted immunoliposomes with high concentrations of the NIRF dye, DY-676-COOH (excitation / emission: 674 nm /699 nm) in the aqueous interior and the green fluorescent phospholipid NBD-DOPE (excitation / emission: 480 nm /530 nm) on the lipid bilayer was reported earlier [23–25]. The liposomes were fused with human FAP’scFv conjugated to micelles to get FAP-IL or murine endoglin scFv conjugated micelles to get mEnd-IL. Human and murine FAP share a high amino acid sequence homology and hence antibody cross reactivity [26], whereas murine and human endoglin share no homology or antibody cross reactivity.

### Uptake of liposomal probes and imaging

To prove the in vitro selectivity of the respective immunoliposomes to human FAP (FAP-IL) or to murine endoglin protein (mEnd-IL), human fibrosarcoma cells stably expressing FAP (HT1080-hFAP) and murine melanoma (B16F10-mEnd) endogenously expressing low levels of endoglin and further stably cloned with a murine endoglin gene were used. 30,000 cells of each cell line were seeded and grown for 16 h on poly-L-lysine-coated 8-well culture slides (BD Biosciences), then treated with 200 nmol (final lipid) of the liposomes for 6 h at 37 °C. The non-targeted quenched liposome, (LipQ) and the free DY-676-COOH (at a concentration equivalent to the dye content of FAP-IL) were used as controls. Likewise, the murine macrophage cell line J774A.1 was seeded at 50,000 cells / well and treated with the respective probes to substantiate their uptake by phagocytosis. Cell harvest, nuclei stain with Hoechst-33258 (Applichem), mounting with Permafluor and subsequent confocal microscopy was done as reported in detail earlier [23]. Whereas the nuclei were visualized with a 405 nm laser diode and a 420-480 nm band pass filter, NBD-DOPE was detected similar to GFP by exciting at 488 nm and capturing the fluorescence at 530 nm. A 633 nm Argon laser was used to excite DY-676-COOH and the emission captured with a 650 nm long pass filter. A 63x magnification was applied for all images.

### Animals

All animal studies were approved by the regional animal committee and conformed to international guidelines on the ethical use of animals. Female athymic nude mice (Hsd:Athymic Nude-Foxn1^nu^ nu/nu; Harlan Laboratories) ranging between 10-18 weeks were housed under standard conditions with ad libitum mouse chow and water. One week before probe injection and imaging, the mice were given a low pheophorbide diet (C1039, Altromin) in order to reduce tissue autofluorescence.

### Determination of the biodistribution of liposomes by fluorescence imaging

**Fig. 1:**
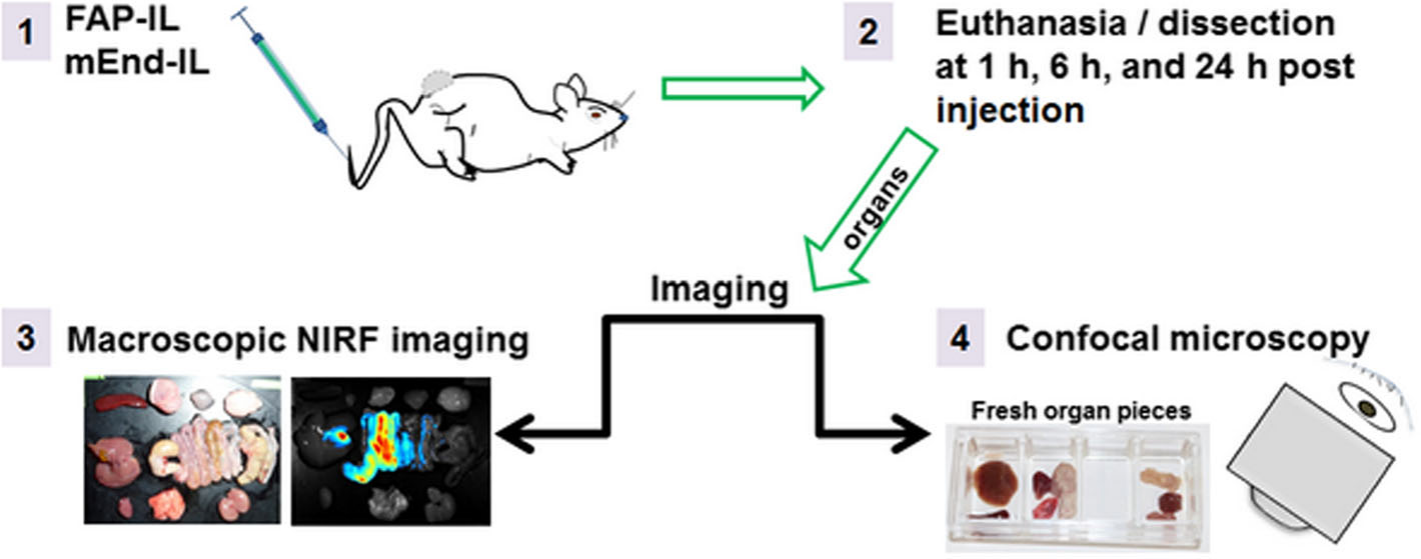
Scheme of the steps involved in determining the biodistribution of FAP-IL and mEnd-IL in mice

A comparison of macroscopic and microscopic imaging of freshly dissected organs was performed according to the following workflow (Fig. 1).

### Macroscopic NIRF imaging and determination of the biodistribution of FAP-IL and mEnd-IL

The mice were anesthetized with 2% isoflurane and the respective probes (20 μmol per kg weight (final lipids) of FAP-IL or mEnd-IL diluted in PBS to 150 μl final volume) were administered by tail vein injection. The animals were sacrificed at different time points post injection (p.i.) and the organs immediately excised and imaged. Macroscopic images of the excised organs were acquired with the Maestro^TM^ in vivo fluorescence imaging system (Cri-InTAS, *Woburn USA*) with filters for the excitation range 615-665 nm and acquiring emission with a cut-in filter (>700 nm). The background autofluorescence were unmixed and determination of the semi-quantitative levels of the fluorescence intensities of respective organs were done with the Maestro software by assigning regions of interest (ROIs) on each of the intensity scaled (for exposure time, camera gain, binning and bit depth) organs as described elsewhere [23]. Fluorescence intensities of the ROIs were derived as average signal (scaled counts/s) and are comparable among each other.

### Euthanasia

Animals were anesthetized with 2% isoflurane till they no longer reacted to touch, and then sacrificed with carbon dioxide till breathing stopped completely.

### Confocal microscopy of freshly isolated organs

Immediately after excision, the organs were quickly rinsed in sterile PBS and placed on the glass platform of an LSM780 confocal microscope (Zeiss, Jena Germany). Otherwise, a small piece of the freshly excised organs was smoothly cut with a sharp scalpel and placed with the smooth surface lying on a glass coverslip of a Lab-Tek™ 4-well borosilicate cover-glass system (Thermo-Scientific, Germany) and imaged on an LSM510Meta confocal laser scanning microscope (Zeiss, Jena Germany). To avoid dehydration during tile scanning microscopy, a drop of PBS was placed in one empty well (Fig. 1) and the chamber slides covered all through microscopy (LSM510Meta). In this constellation, whereby the organs are imaged without processing, the strong tissue autofluorescence (blue to green fluorescence) of the fresh organs permits detail visualization of the organ structures, whereas the injected contrast agents with fluorescence absorption and emission maxima beyond the autofluorescence range (e.g. liposomal DY-676-COOH: abs/em. 674 /699 nm) can be clearly distinguished. Images were acquired at similar excitation and emission settings like in cellular uptake experiments. The whole cells were visualized based on autofluorescence by excitation in the blue region with a 405 nm laser diode and a 420-480 nm bandpass filter. The fluorescence of minimal green autofluorescent tissue components and also liposomal NBD-DOPE was detected with the GFP filter at 530 nm after excitation at 488 nm. DY-676-COOH was excited with a 633 nm argon laser and emission captured with a 650 nm longpass filter. Images were acquired at a 20x magnification.

### Statistical data

The student’s *t*-test was used, if not otherwise stated, to deduce the level of significance, when normality and equal variance tests were passed. If not, Mann-Whitney-Rank sum test was applied. All experiments were done at least twice. For animal trials, four or more animals / group were used. Differences resulting in *P* < 0.05 were considered significant.

## Results

### Properties of the immunoliposomes

**Fig. 2:**
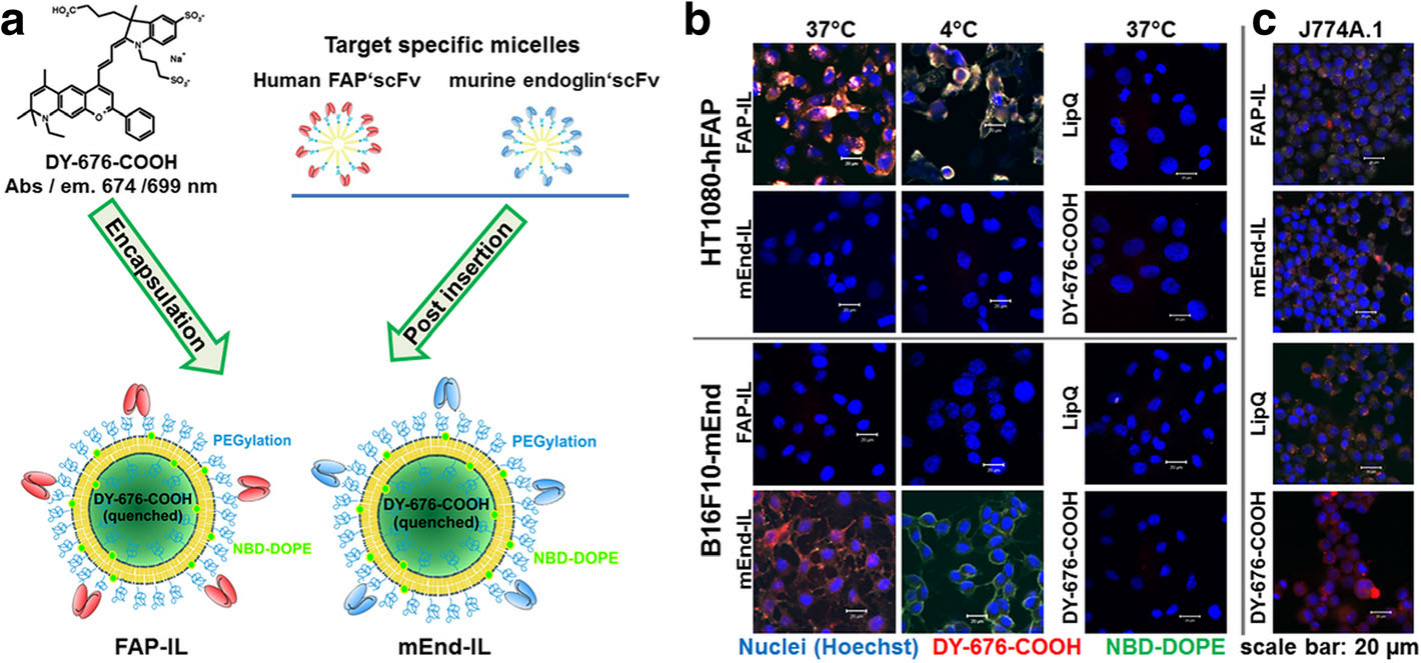
Schematic presentation of the properties of the immunoliposomes used. **a** Preparation of immunoliposomes with high concentration of encapsulated DY676-COOH, and post insertion of micelles bearing human FAP and murine endoglin scFv. **b** Target selectivity of FAP-IL, mEnd-IL, non-targeted quenched liposome, LipQ and the free DY-676-COOH (at a concentration equivalent to the DY-676-COOH content in FAP-IL) after incubation with target expressing cells at 37 °C or 4 °C for 6 h. **c** Validation of the phagocytic uptake of liposomal probes by murine macrophages J774A.1 after 6 h incubation at 37 °C

The immunoliposomes contained high concentrations of the fluorescence-quenched NIRF dye, DY-676-COOH encapsulated in their aqueous interior and the non-quenched green fluorescent NBD-DOPE on the lipid bilayer. Hence, the quenched liposomes termed LipQ possessed dual fluorescence for imaging in the green and also near-infrared wavelength range. The green fluorescent phospholipid enables detection of the intact liposomes prior to their degradation and activation of the DY-676-COOH. For selective targeting, single chain antibody fragments directed to the human FAP or murine endoglin proteins were conjugated to their surface by the post-insertion method (Fig. 2a) and the probes termed FAP-IL and mEnd-IL, respectively. Mouse and murine FAP share high amino acid sequence homology and subsequent antibody cross-reactivity, whereas murine endoglin antibodies do not cross react with human endoglin and vice versa. Consequently, fibrosarcoma cells expressing endogenous human endoglin, and stably transformed to additionally express human FAP selectively took up FAP-IL, but not the mEnd-IL, control LipQ nor the free DY-676-COOH (Fig. 2b, *HT1080-hFAP*). Likewise, the mouse melanoma cell line with high levels of stably transformed murine endoglin selectively took up mEnd-IL, but not FAP-IL, LipQ nor the free DY-676-COOH (Fig. 2b, *B16F10-mEnd*), which substantiates the target selectivity of the respective immunoliposomes. In the endoglin expressing cell line in particular, binding of mEnd-IL at 4 °C reveals only the non-quenched green fluorescent phospholipid embedded in the liposomal bilayer (Fig. 2, *B16F10-mEnd, 4 °C*), underscoring the importance of the phospholipid in tracking the intact quenched liposomes prior to their activation. Furthermore, all the probes could be taken up by phagocytosis, as can be seen in the murine macrophage cell line J774A.1 (Fig. 2c).

### Macroscopic NIRF imaging show fluorescence distributions indicative of first pass effect, retention and also washout at different time points post injection

We verified whether the first pass effect of fluorescent probes can be effectively imaged and whether the data acquired would provide indications of the probes’ later degradation and clearance from the system. Also, the selectivity of the immunoliposomes for their target proteins was investigated. Provided there is overexpression of FAP or endoglin proteins in any mice organs, selective accumulation and retention of the respective probes will take place, owing to the ability of both probes to bind the murine targets. The probes were therefore intravenously applied in nude mice and the organs isolated at 1 h, 6 h and 24 h post injection (p.i.) and subjected to NIRF imaging.

**Fig. 3:**
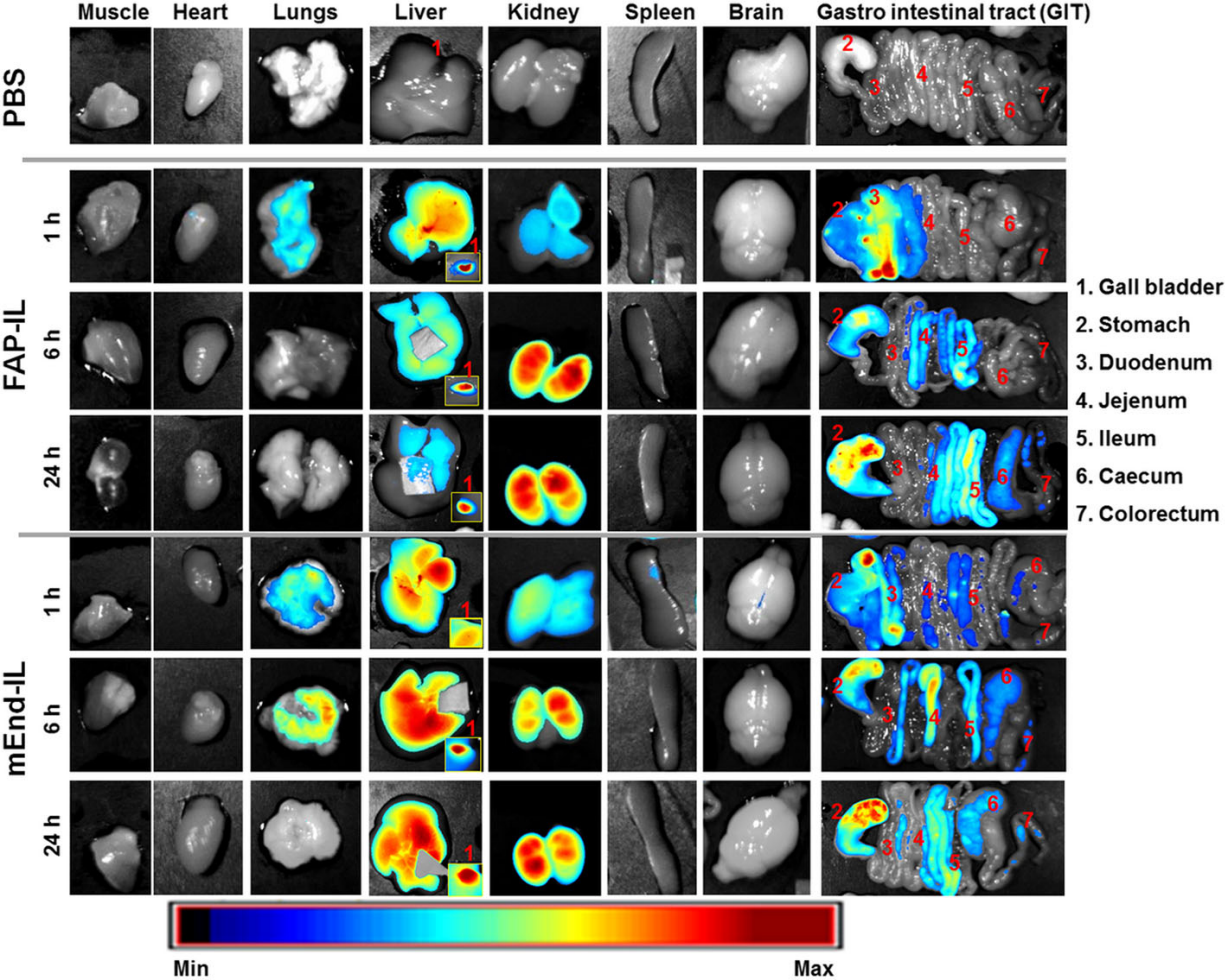
Representative intensity scaled NIRF images of mice organs showing distribution of liposomal fluorescence at different time points post injection. At 1 h post injection first pass distribution in lungs, kidneys, liver, gall bladder and preliminary release to the duodenum are seen for both FAP-IL and mEnd-IL. At 6 h post injection, FAP-IL is washed out from the lungs and partially from the liver and secreted to the gall bladder, but retained in the kidneys. Release from the gall bladder to the duodenum and excretion via the GIT is evident in the fluorescence movement from duodenum to the jejunum, ileum and colorectum with time. The mEnd-IL is retained in the lungs, kidneys and liver and only gradually eliminated via the GIT with time

At 1 h p.i., high fluorescence signals were detected in the lungs, liver, kidneys, gall bladder and duodenum (Fig. 3). This represents probe distribution resulting predominantly from the first pass effect. After intravenous injection, the probes rapidly circulate in blood and are retained or visible in organs with high levels of blood flow (e.g. lungs) or high levels of blood flow as well as processing (e.g. liver). Due to initial rapid degradation by liver kuppfer cells and secretion to the bile, a high fluorescence is detected in the gall bladder at this time point (1 h p.i.). From the gall bladder the probes are further released to the duodenum, as seen in the high fluorescence of the duodenum. Evident for a partial elimination of the probe via the kidney, a minimal fluorescence signal is seen in the kidneys at 1 h p.i. (Fig. 3). Comparing FAP-IL and mEnd-IL, some differences can be seen in the fluorescence intensity of the organs at various time points. While the FAP-IL causes lung fluorescence only as a “first-pass” effect seen at 1 h p.i., the mEnd-IL accumulates and is retained in the lungs for longer durations (Fig. 3, *mEndl-IL 6 h*). Likewise, FAP-IL in the liver is rapidly degraded and eliminated with time, whereas a lower amount is retained in the kidneys and persists till 24 h. In the gastro intestinal tract (GIT), FAP-IL fluorescence is seen to move over time, from the duodenum towards jejunum, ileum and caecum from where it is excreted through the colorectum in feces.

Opposed to FAP-IL, the mEnd-IL signals increases in the liver and GIT till 24 h p. i. The liver seems incapable of rapidly degrading the mEnd-IL, hence very high liver fluorescence is seen at 24 h. Furthermore, the gall bladder from mice which received mEnd-IL show increasing fluorescence signals with time, and relatively high levels at 24 h p.i. These observations strongly suggest that accumulation of mEnd-IL in the liver and other organs such as lungs are not a result of the first pass effect alone, but rather based on molecular interaction with murine endoglin protein which is possibly expressed in these organs at high levels. The differences observed between the FAP-IL and the mEnd-IL which only differ in the targeting moieties used, therefore raised many questions. It was thus vital to pinpoint the subcellular factors responsible for the different biodistribution and retention.

**Fig. 4:**
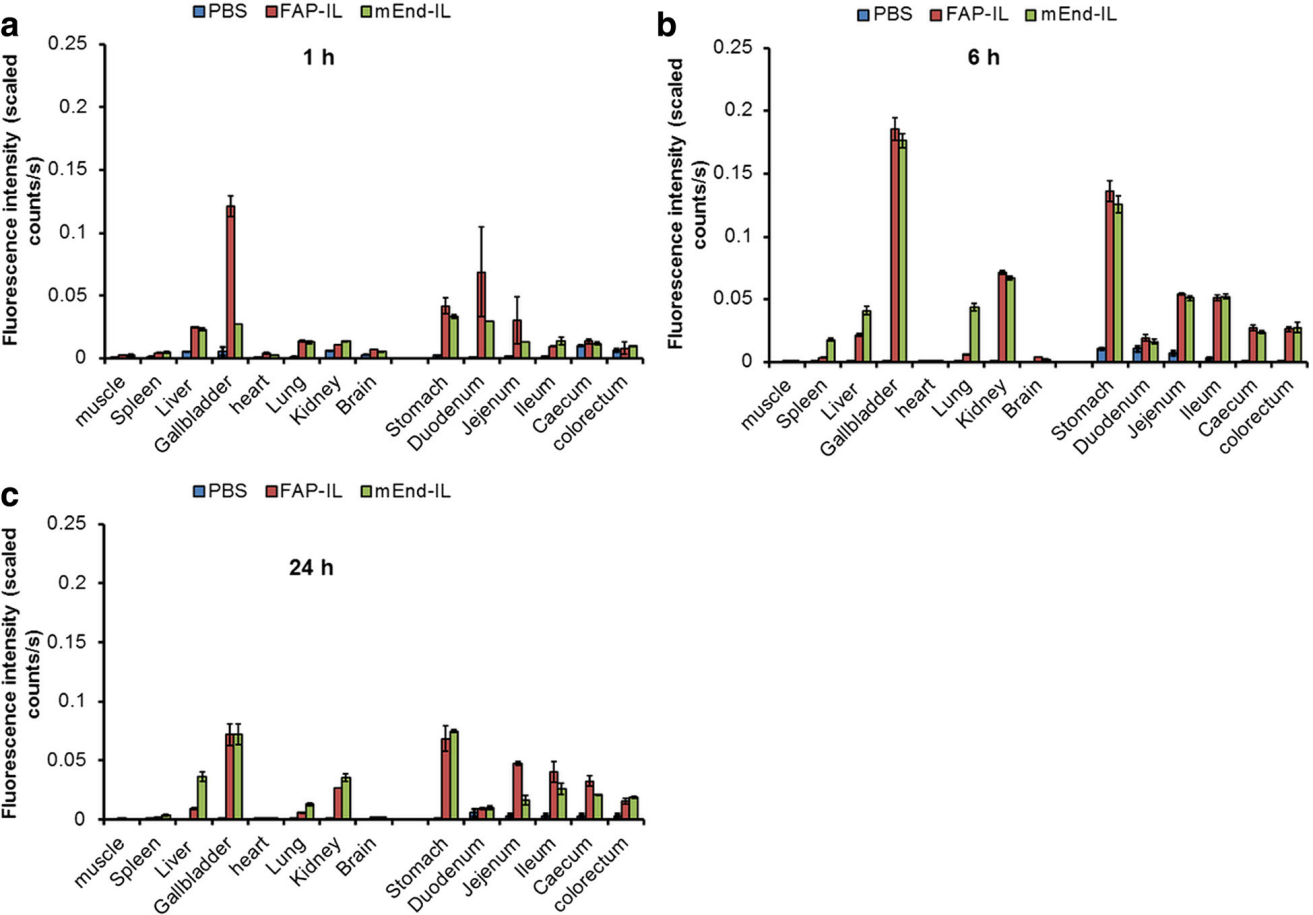
Semi-quantitative levels of fluorescence intensities of organs at the given time points (**a** 1 h, **b** 6 h and **c** 24 h) post injection of PBS, FAP-IL or mEnd-IL. Each bar depicts the mean fluorescence of the respective organs per time point. n = 4 ± SD for the 6 h and 24 h groups and n = 3 ± SD for the 1 h group

Semi-quantitative evaluations revealed fluorescence intensities of organs which correlated well with the observations made earlier (Fig. 3), and substantiated the distribution and subsequent elimination of the probes with increase in duration post i. v. injection. Thus, the highest fluorescence levels were deduced 6 h p.i. (Fig. 4).

### Confocal microscopy of freshly resected lungs, liver and kidneys pinpoint the subcellular components involved in probe elimination or retention

**Fig. 5:**
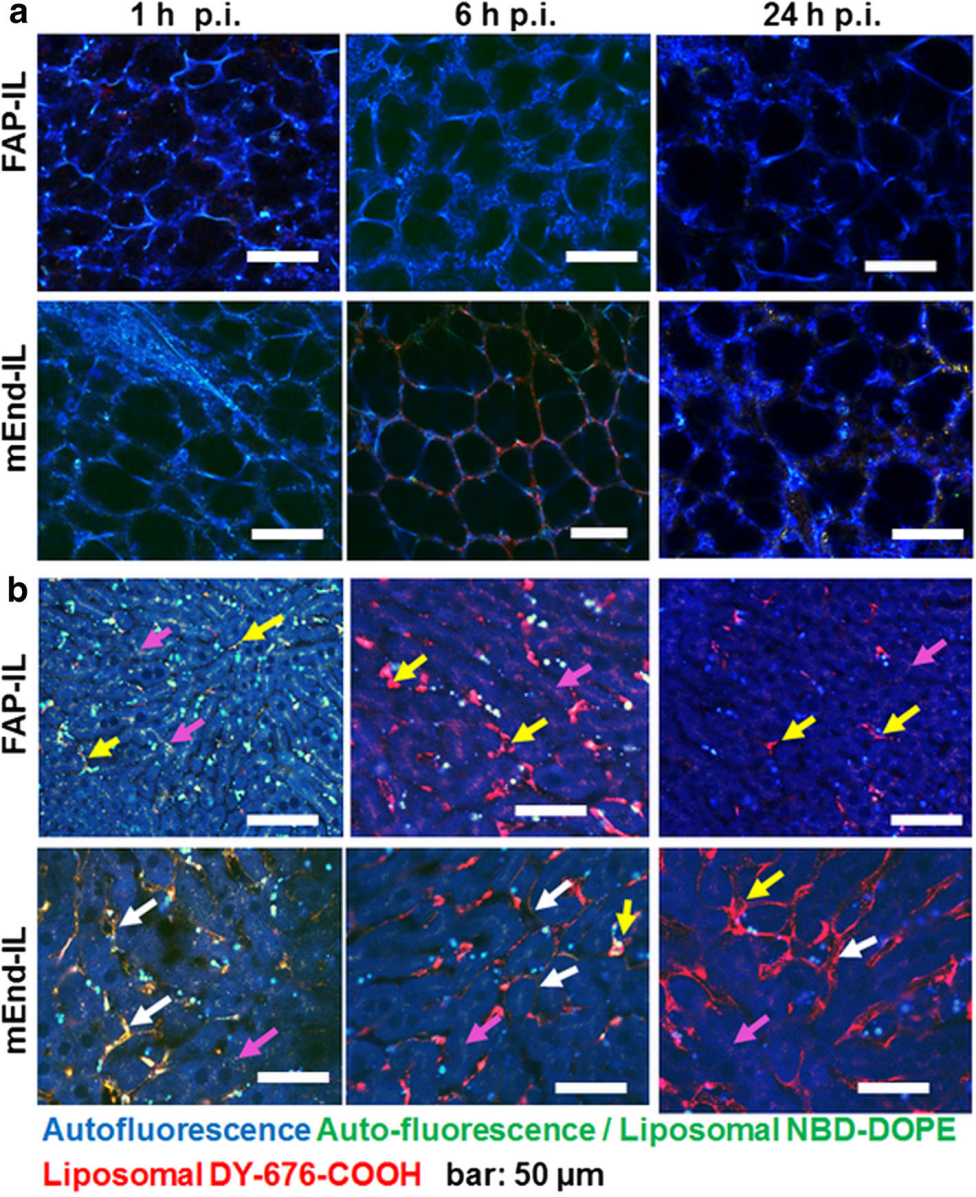
Subcellular distribution of FAP-IL and mEnd-IL fluorescence in freshly resected lungs and liver. **a** Lungs, showing the liposomal *green*/*red* fluorescence particularly 6 h post injection of mEnd-IL. **b** Liver, showing the liposomal *green*/*red* fluorescence of FAP-IL in kupffer cells (*yellow arrows*) and bile canaliculi (*pink arrows*) and the mEnd-IL fluorescence predominantly in the liver sinusoidal cells (*white arrows*). At 6 h and 24 h post injection the location of the liposomal fluorescence is the same, but a predominant red fluorescence of the released DY-676-COOH is seen

Lung tissues of mice isolated 1 h or 6 h p.i. of mEnd-IL revealed high fluorescence signals of the endothelia cells (Fig. 5a). Likewise, the liver sinusoidal cells showed strong fluorescence of the mEnd-IL at these time points (Fig. 5b, *white arrows*), whereas very few kuppfer cells were detected with the mEnd-IL fluorescence (Fig. 5b, *yellow arrows*). Furthermore, the strong mEnd-IL based fluorescence in the endothelia cells only reduced gradually with time. Thus, at 24 h p.i. of mEnd-IL, liver endothelia cells still showed high fluorescence signals. Opposed to mEnd-IL, the liver excised from mice which received FAP-IL revealed liposomal fluorescence predominantly in the kuppfer cells (Fig. 5b, *yellow arrows*) and the bile canaliculi (Fig. 5b, *pink arrows*). This was high at 1 h and 6 h p.i., but rarely detectable after 24 h. Also, the lungs showed mild liposomal fluorescence 1 h post application of FAP-IL, but revealed no further signals at 6 h and 24 h post injection, indicating that their accumulation in lungs is due to the first-pass effect.

**Fig. 6:**
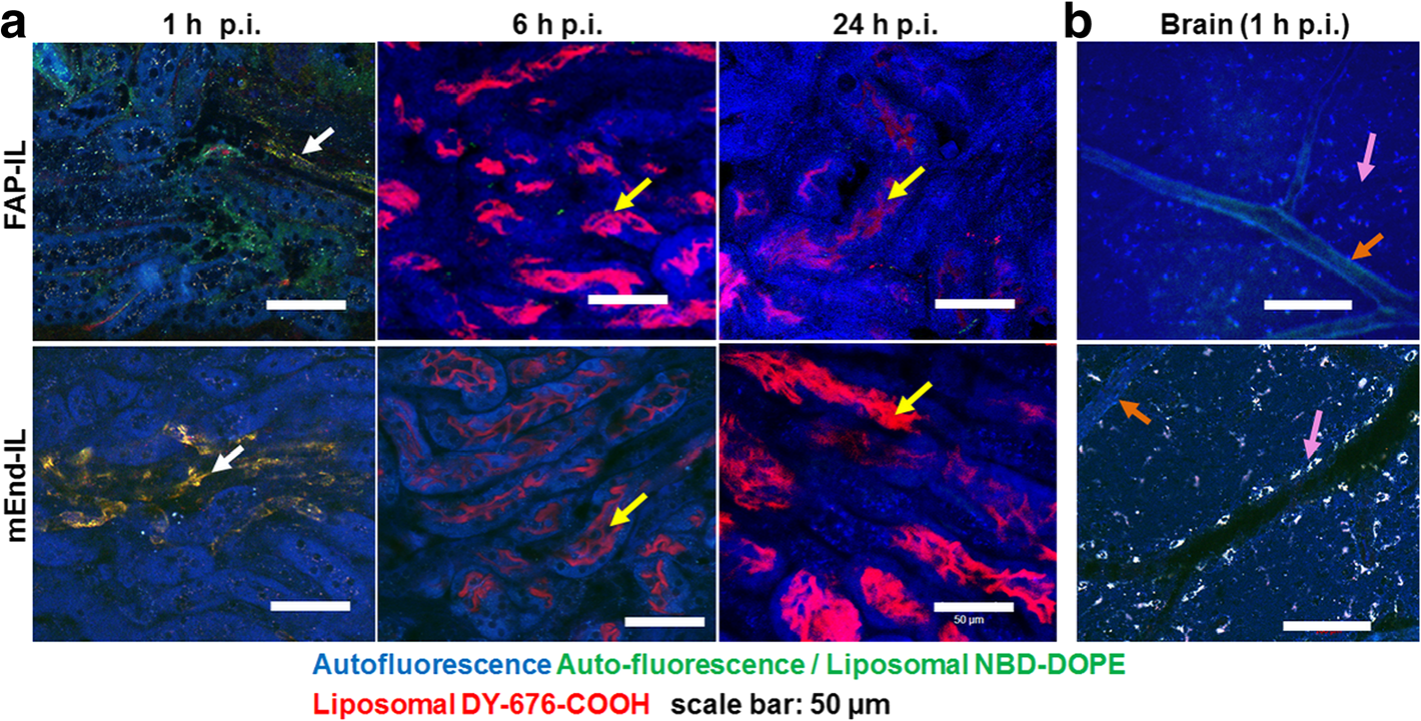
Subcellular distribution of FAP-IL and mEnd-IL fluorescence in freshly isolated kidneys (**a**) and brain (**b**). The liposomal signals are seen as *green*/*red* fluorescence in the kidney cells (FAP-IL) or blood vessels (mEnd-IL) at 1 h post injection (*white arrows*). At 6 h and 24 h p.i., a predominant *red* fluorescence of the released DY-676-COOH is seen in the tubules of the kidney pyramids (*yellow arrows*). **b** Opposed to FAP-IL which shows no signals in the brain, mEnd-IL accumulates in cells lying close to the brain blood vessels (*pink arrows*), whereas no signals are seen in the arteries (*orange arrows*)

Interestingly, the kidneys revealed comparable abilities to retain both the mEnd-IL and the FAP-IL fluorescent components for longer durations after injection. However, the localization of the characteristic DY676-COOH fluorescence in the kidneys over time exposed indications for its mode of elimination. Thus, microscopic images of kidney cross sections showed fluorescence localized in the cortex at 1 h and 6 h p. i. of both probes (Fig. 6a). Additionally, the mEnd-IL based fluorescence was seen in the blood vessels of the kidneys, 1 h after injection (Fig. 6a, *mEnd-IL white arrows*). At 24 h post application, this fluorescence was predominantly localized in the tubules of the kidney pyramids and pelvis, irrespective of the probe applied.

Though organs like the spleen, heart and brain retained little or none of the probes injected, their morphological detection based on autofluorescence was possible. In the brain, the arteries and veins showed different levels of autofluorescence (Fig. 6b). Compared to FAP-IL, the mEnd-IL seems to partially bind the brain endothelia cells. This was evident in a minor fluorescence of the cells lining the blood vessels in the brain (Fig. 6b, *mEnd-IL pink arrow*). Opposed to this, FAP-IL based fluorescence was not seen in the brain. Compared to the brain, there was no fluorescence of the probes detected in the heart, whereas the spleen revealed distorted fluorescence distribution of especially the mEnd-IL (not shown).

### Influence of post mortem staining of nuclei in fresh organs directly after isolation

#### Microscopic imaging: heart, lungs, spleen, liver, kidneys and tumors

We also verified if detection of internalized probes will be possible after rapid nuclei stain of the fresh organs. We observed that imaging based on the tissue autofluorescence was superior to Hoechst-33258-stained fresh tissues. This was basically because the morphological features of the tissues and organs were lost, due to a strong unspecific stain of several tissue components by the Hoechst-33258 solution (results not shown). This observation further underlines the benefits of exploiting the tissue autofluorescence in microscopic determination of the biodistribution of contrast agents.

## Discussion

In most preclinical biodistribution studies, researchers generally assume that the accumulation of drugs or fluorescent probes in the liver or kidneys portray their degradation and excretion via the hepatobiliary or urinary routes [12, 23]. In such studies organs are analyzed at a single time point (e.g. 6 h or 8 h) post probe application. However, vital information on the subcellular distribution and possible damage to the organs due to long retentions remain undocumented in such studies. The underlying results demonstrates the importance of monitoring the biodistribution at many time points in order to make reliable conclusions on probe distribution, retention and their later elimination from the system. A longer retention of some contrast agents in several organs could result in adverse side effects [13, 14, 27] depending on the subcellular localization, since different tissue cells react differently to different agents. Thus, it is important to know which subcellular factors are responsible for the retention of probes in different organs, for example excretory organs such as the liver, kidney and also sensitive organs not involved in biodegradation and excretion, such as the lungs, brain and heart. A longer retention in the liver could be due to a slower degradation, or unwanted affinity of the probes to molecular structures in the organs as seen with the mEnd-IL. Based on the contrast agent or therapeutic drug in question, this may cause adverse side effects. For example prolonged retention in the liver may implicate adverse effects of the probes on the kuppfer cells, or sinusoids of the liver or on the secretion to the bile. Likewise, longer retentions in the kidney due to formation of unfilterable aggregates may pose damages with time. A major reason why many researches do not include microscopic validation of the subcellular localization of probes is lack of suitable equipment. Thus, we verified if the use of simple and easily accessible microscopy setups could enable this.

Using macroscopic NIRF imaging, we could determine the biodistribution of targeted immunoliposomes based on the first-pass effect post injection and also their retention based on molecular targeting to different target cells. Interesting differences were seen between the FAP-IL and the mEnd-IL. Whereas, the FAP-IL distributed predominantly based on the first pass effect and subsequent elimination, the mEnd-IL revealed longer durations of retention in vital organs such as the lungs, liver and kidneys. The first-pass effect of FAP-IL was especially characterized by fluorescence of lungs which disappeared before 6 h post injection. This was coupled with a preliminary fluorescence of the gall bladder, liver, duodenum and kidneys at 1 h p.i., which increased in these organs after 6 h, and subsequently decreased 24 h after injection. Furthermore, a gradual movement of the fluorescence from the duodenum towards the jejunum, ileum and colorectum with increasing time post application indicated probe elimination via feces. The relatively high fluorescence signals of both immunoliposomes seen in the stomach could not directly be explained. We postulated previously [25] that this fluorescence may result from pancreatic and partial bile release of probes into the stomach like in humans, or due to reflux from the duodenum. This is supported by the fact that the fluorescence increases with time post injection and is very high even at time points when there is no fluorescence in the duodenum. Although the liposomes are not pH sensitive, and microscopic images demonstrate the secretion of the liposomal DY-676-COOH and NBD-DOPE in the bile canaliculi of the liver, which implies their eventual delivery to the gall bladder and stomach as individual components and not intact liposomes, the low pH of the stomach could possibly influence the DY-676-COOH and play a role in the high NIRF detected here. Contrary to FAP-IL, the combination of first-pass effect and molecular targeting by the mEnd-IL caused longer retention of the probe in many organs including the lungs, liver and kidneys as seen by macroscopic imaging. This retention could be detected based on the different time points considered and exposes the relevance of this consideration in biodistribution studies. Besides considering several time points, it is also important to pinpoint the sub cells responsible for the probe retention.

We therefore implemented a simple fresh organ microscopy setup to validate this. Although tissue autofluorescence interferes with in vivo fluorescence imaging, it has been exploited in defining several organs / tissue structures and to distinguish pathological changes in diseased tissues [16]. Thus, cellular and tissue autofluorescence originating from mitochondria, lysozymes, lipo-pigments and pyridinic (NADPH), flavin coenzymes, collagen, elastin, hemoglobin and melanin are successfully exploited for diverse applications such as in endoscopic imaging [28] and intravital microscopy [29, 30]. These tissue fluorophores absorb and emit light at different wavelengths which lie beyond the near-infrared optical window (650 nm - 900 nm) [17]. Consequently, the fluorescence of the liposomal encapsulated NIRF dye, DY-676-COOH (abs/em max. 674 /699 nm) could be easily distinguished from tissue autofluorescence of freshly excised organs. Whereas the lungs of FAP-IL treated mice revealed no detectable fluorescence signals, the liver and kidneys revealed distinct liposomal fluorescence at the different time points investigated. The kidneys showed mild fluorescence of the blood vessels and tubules of the cortex at 1 h p.i. and predominantly in the pyramids and pelvis with increased duration post injection. This indicates a partial, but gradual elimination of the probes in urine [25]. It was shown previously that DY-676 is highly hydrophobic and hence preferentially eliminated by the hepatobiliary route [12]. Consistent with this, the FAP-IL based fluorescence was located predominantly in kuppfer cells of the liver at all the investigated time points. The liver kuppfer cells are responsible for the host defense. When toxic or foreign substances are recognized by the system they opsonize the foreign substances, making them recognizable by macrophages which engulf them in the blood and migrate to the liver. In the liver the macrophages (both infiltrating and resident) are called kuppfer cells [31]. While in the liver they degrade the foreign substances which can then be secreted to the bile for elimination to the duodenum as was evident in the macroscopic images. However, the probes can also reach the liver directly through blood circulation. This is achieved by the first pass effect as well as repeated circulation of long circulating probes such as the immunoliposomes used herein. Notably, the FAP-IL undergoes only circulation, phagocytic uptake and degradation due to the lack of targets in the mice used, since FAP is exclusively expressed in diseased but not healthy tissues [21].

Opposed to FAP-IL, the mEnd-IL was detected in the lung, brain, kidney and liver endothelia cells. The fact that mEnd-IL localized in these endothelial cells substantiates the stability of the PEGylated liposomes in the blood circulation and their selectivity for murine endoglin. Hence, they enter the liver and other organs as intact vesicles which then specifically bind and are taken up and degraded by the respective endothelial cells, releasing the encapsulated DY-676-COOH. The free DY-676-COOH can be taken up by phagocytic cells but not by other cell types (see also Fig. 2), and is more rapidly eliminated than the long circulating liposomes in vivo [24]. Thus, the predominant green fluorescence and co-localization of the green and red fluorescence of liposomal NBD-DOPE and DY-676-COOH in organs such as liver and kidneys at 1 h p.i. for example, is indicative of the accumulation of intact liposomes, which eventually get degraded to release / activate the encapsulated DY-676-COOH in these organs (see Fig. 5b and Fig. 6a). This highlights the role of the non-quenched green fluorescent phospholipid, NBD-DOPE in tracking the intact versus degraded liposomes as was demonstrated in time course experiments earlier [24, 25] and also herewith (Fig. 2b, *4 °C*). Based on earlier cytoxicity studies [19], it is known that the encapsulated DY-676-COOH is not cytotoxic. Considering that a cytotoxic substance used in its place could exert damages on the liver, lungs and brain endothelial cells upon long retentions, our results expose the importance of including microscopy as part of a biodistribution study. Furthermore, our results show that this is possible on freshly resected tissue. Thus, exploiting the tissue autofluorescence helps detect morphological changes that may result from adverse effects of the applied probes. This is especially feasible when characterizing the biodistribution of contrast agents coupled to a fluorophore with absorption and emission maxima in the near infrared optical window. Nevertheless, fluorescent dyes with lower wavelengths can be detected if their concentrations are much higher than that of the tissue autofluorescence. In our images for example, we could detect the green fluorescent phospholipid, NBD-DOPE (abs / em.: 480 nm / 530 nm), due to its high concentration and resulting strong signals which outshined the background autofluorescence. We therefore strongly recommend using this method when analyzing fluorescent probes with spectroscopic properties lying beyond the tissue autofluorescence range (650-900 nm). However, if the concentration of a probe is very high it could be detected similar to the NBD-DOPE used herein. In this case a comparison with a second dye would be of advantage, to avoid false interpretation.

## Conclusion

We demonstrate a simple, cost effective approach to suitably determine the subcellular distribution of contrast agents and drugs in freshly isolated organs by confocal microscopy. The result sheds light on the cellular subsets involved in the biodistribution of contrast agents and suggests the consideration of several time points in biodistribution studies. Implementing the approach will improve preclinical characterization of contrast agents or theranostic agents designed for future clinical applications. Furthermore, the approach holds potentials to be expanded to clinical units where fluorescence based intraoperative setups and biochemical / histological evaluation of excised tissues are microscopically characterized immediately. We are therefore convinced that many researchers and clinical units will benefit from the simple approach demonstrated herein.

## Abbreviations

FAP: Fibroblast activation protein
NIRF: Near infrared fluorescence
p.i.: Post injection
scFv: Single chain variable fragment
